# Successful Treatment of Bilateral Renal Mucormycosis With Isavuconazole: A Case Report

**DOI:** 10.7759/cureus.42219

**Published:** 2023-07-20

**Authors:** Yaseen Najjar, Shirley Botros, Emily Acker, Ananthakrishnan Ramani, Kelly Beers

**Affiliations:** 1 Internal Medicine/Nephrology, Albany Medical Colloge, Albany, USA; 2 Internal Medicine Albany, Albany Medical College, Albany, USA; 3 Infectious Diseases, Albany Medical College, Alban, USA; 4 Nephrology, Albany Medical College, Albany, USA

**Keywords:** hd ( hemodialysis ), intravenous drug use (ivdu), type i diabetes mellitus, isavuconazole, isolated renal mucormycosis, rhizopus oryzae

## Abstract

Isolated renal mucormycosis (IRM) is a rare disease with high mortality, more commonly seen in immunocompromised patients. Management has traditionally included antifungal drugs with or without nephrectomy. We present the case of a 34-year-old female with a past medical history of type 1 diabetes mellitus and intravenous heroin use who presented with fever, flank pain, hematuria, and vomiting. She was found to have an oliguric acute kidney injury (AKI) with a serum creatinine (Cr) of 2.5 mg/dL. CT showed bilateral emphysematous pyelonephritis and ureteral cultures grew Rhizopus species. Amphotericin B was started before being switched to isavuconazole due to worsening AKI, and hemodialysis was only required transiently. Rather than the traditional approach to treatment, a conservative approach that preserved kidney function was utilized, and the patient was successfully treated with six months of isavuconazole.

## Introduction

Rhizopus and Mucor are filamentous fungi that are found in soil, plants, and decayed fruits. Rhizopus can be distinguished from Mucor by its rhizoids and stolons and its formation of black cotton-like colonies [1]. Both organisms cause similar disease patterns and share infectious characteristics and treatments. The organisms can be contracted mainly by inhalation of spores into the paranasal sinuses and lungs or direct contact with the skin [2]. Infection is rapidly destructive and spreads quickly to adjacent structures, such as the orbits and cerebrum, in the case of sinus infection. The fungus can also invade blood vessels, which is known as angioinvasion, causing fungemia and subsequent involvement of other organs such as the kidneys. Infections are typically more severe in immunodeficient hosts. Diabetes and ketoacidosis are classic risk factors for this devastating infection, as are hematological malignancies and intravenous (IV) drug abuse. Due to angioinvasion, surgical debridement (e.g., nephrectomy in the case of kidney involvement) is crucial to clearing the infection. Moreover, these fungi are resistant to the usual azoles and echinocandins and only sensitive to amphotericin B and newly developed triazoles [3].

## Case presentation

**Figure 1:**
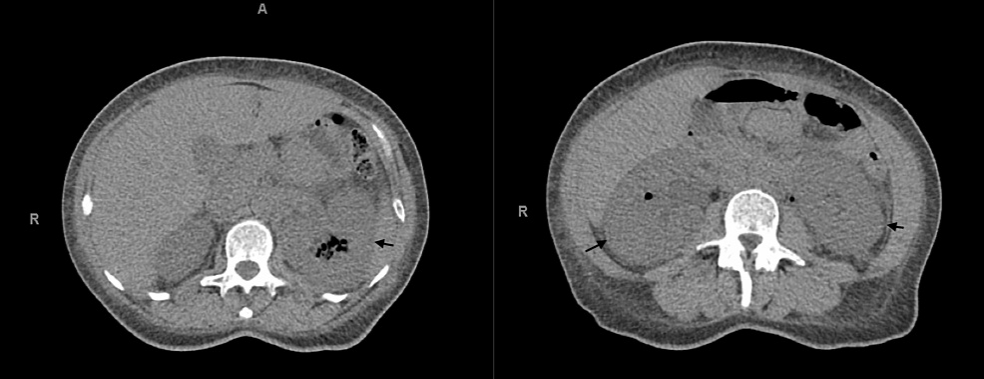
Non-contrast axial CT of abdomen and pelvis showing bilateral emphysematous pyelonephritis. Bilateral kidneys are enlarged with multiple foci of air in the bilateral kidneys, left greater than right.

A 34-year-old female with a past medical history of uncontrolled type 1 diabetes mellitus and IV drug use on methadone, with recent IV heroin use, was transferred to our tertiary medical center for management of emphysematous pyelonephritis. Ten days before transfer, she was diagnosed with right-sided emphysematous pyelonephritis on a CT scan at an outside hospital emergency department. The patient refused admission and was therefore treated as an outpatient with oral amoxicillin/clavulanate. Six days later, her condition worsened, prompting her to return to the same hospital with fevers, nausea, vomiting, hematuria, bilateral flank pain, and oliguria with a serum Cr of 2.5 mg/L. Non-contrast CT showed bilateral emphysematous pyelonephritis with persistent changes in the right kidney and new changes in the left (Figure 1). Two days later, she was transferred to our medical center for further treatment given her oliguria and rising Cr.

Initial labs on admission are shown in Table 1. Broad-spectrum empiric antibiotics were started, with the patient receiving one dose of IV meropenem and one dose of IV vancomycin before being treated with five days of IV piperacillin-tazobactam. A cystourethroscopy was emergently performed for bilateral ureteral stent placement, and intra-procedural findings were notable for black sludge upon dilation of the right ureteral orifice, which was collected and sent for culture. While urine and blood cultures were negative, aerobic cultures from the right ureter grew filamentous fungi, with preliminary identification as zygomycete and final speciation as Rhizopus. Anaerobic culture from the right ureter produced two colonies of *Finegoldia magna*. Amphotericin B liposomes were added to the antimicrobial regimen. Subsequently, piperacillin-tazobactam was changed to ampicillin coverage of *F. magna* to complete a course of 10 days of beta-lactams. Blood cultures remained negative repeatedly throughout the patient’s admission. Her clinical picture was complicated by worsening oliguria, hypervolemia, and hyperkalemia, with Cr peaking at 8.37 mg/L, which was attributed to the nephrotoxic effects of amphotericin B. After six days of amphotericin B, it was switched to isavuconazole in an effort to preserve the remaining kidney function. Hemodialysis was initiated, and after only two sessions, hyperkalemia resolved and urine output started to improve. Initially, bilateral nephrectomy was considered in consultation with urology and infectious disease for source control but was deferred upon discussion with nephrology as there was no evidence of abscess or kidney infarction on imaging and the patient was showing signs of clinical improvement. Cr improved to 1.5 mg/dL at the time of discharge, and she was discharged with a plan for two months of oral isavuconazole and 10 days of amoxicillin with infectious disease and nephrology outpatient follow-up.

**Table 1:** Initial laboratory results MCH: mean corpuscular hemoglobin, MCV: mean cell volume, HCT: hematocrit, AST: aspartate aminotransferase, ALT: alanine aminotransferase, ALP: alkaline phosphatase, MRSA: methicillin-resistant Staphylococcus aureus polymerase chain reaction, AFB: acid-fast bacillus

Test	Value	Reference range/units
Hematology		
WBC	17.0	(4.0–9.0) 10 × 3/µL
Hemoglobin	9.3	(13.6–16.7) g/dL
HCT	27.1	(40.0–49.0) %
MCV	88.0	(82.3–93.2) fL
MCH	30.2	(27.8–31.9) UUG
Platelet	138	(130–350) 10 × 3/µL
Neutrophils (%)	14.9 (87%)	(1.6–6.2) 10 × 3/µL (41–67) %
Lymphocyte (%)	1.3 (8%)	(1.1–3.9) 10 × 3/µL (28–42) %
Basophil (%)	0.0 (0%)	(0–1) %
Eosinophil (%)	0.0 (0%)	(0.0–0.5) 10 × 3/µL (0–5) %
Monocyte (%)	(4%)	(4–8.5) %
Chemistry		
Na	123	(135–145) MEQ/L
Cl	89	(99–109) MEQ/L
K	4.7	(3.4–5.2) MEQ/L
Bicarbonate (CO_2_)	21	(21–30) MMOL/L
BUN	36	(7–22) MG/DL
Cr	4.84	(0.80–1.40) MG/DL
Calcium	7.8	(8.6–10.3) MG/DL
Phosphate	3.3	(2.4–4.7) MG/DL
Magnesium	1.8	(1.5–2.6) MG/DL
Albumin	2.4	(3.5–5.2) GM/DL
Bilirubin	0.2	(0.1–1.2) MG/DL
ALP	571	(30–115) IU/L
AST	34	(5–45) IU/L
ALT	32	(5–60) IU/L
Microbiology		
Urine culture	Negative	Negative
Blood culture, 2-set	Negative	Negative
MRSA PCR	Negative	Negative
AFB, sputum	Negative	Negative
Urinalysis		
Protein	2+	Negative
Leukocyte esterase	3+	Negative
Nitrite	Negative	Negative
Hemoglobin	3+	Negative
Glucose	Trace	Negative
Ketones	Negative	Negative
pH	7.0	4.5–7.8
SG	1.010	1.002–1.029
Color	Red	Yellow
Appearance	Cloudy	Clear
Bilirubin	Negative	Negative
WBC	Numerous	0–5/HPF

**Figure 2:**
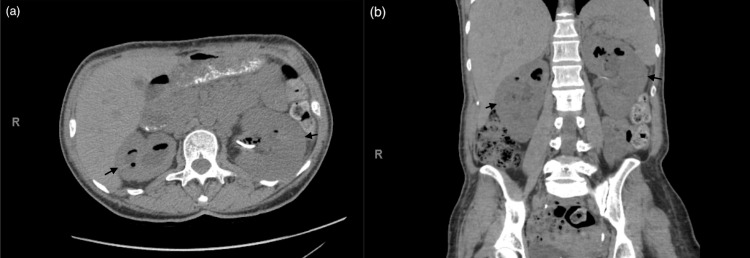
Non-contrast axial (a) and coronal (b) CT of abdomen and pelvis showing recurrence of bilateral emphysematous pyelonephritis.

**Figure 3:**
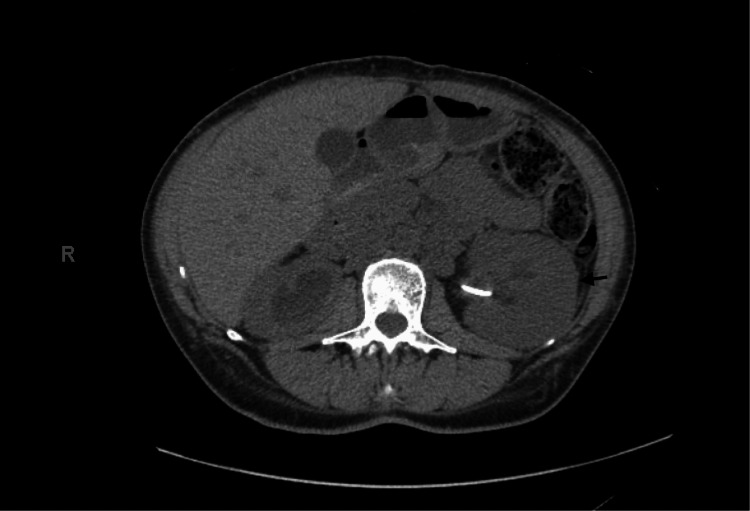
Non-contrast axial CT of abdomen and pelvis showing improvement in bilateral emphysematous pyelonephritis after one week of intravenous isavuconazole.

After treatment, the patient faced several sequences, including recurrent pyelonephritis due to medication nonadherence, acute kidney injury (AKI) requiring dialysis, and bilateral hydronephrosis requiring DJ (Figure 2). After one month, she was re-admitted and restarted on IV isavuconazole. After an improvement was seen on a CT scan within one week (Figure 3), she was discharged with six months of oral isavuconazole. She presented again the following month with similar symptoms, was found to have malpositioned ureteral stents, and underwent bilateral ureteral stent exchange. Cultures from urine from the left ureter grew one colony of group B beta-hemolytic streptococci and Rhizopus arrhizus, while aerobic cultures grew Prevotella bivia. She was restarted on isavuconazole, which she had not been compliant with, and also treated with Levaquin in the hospital. Four months after her initial presentation, the patient presented for the third time with severe AKI requiring renal replacement therapy secondary to obstructive nephropathy. Her course was complicated by hyperkalemia and cardiac arrest, which were successfully revived by CPR. After hemodynamic stabilization, bilateral percutaneous nephrostomy tubes were placed to resolve AKI. Urine cultures from the right nephrostomy grew Rhizopus species during this admission, while blood cultures remained negative. The patient required intermittent hemodialysis during her 10-day admission. She had many subsequent admissions due to flank pain or leakage around her percutaneous nephrostomy tubes; during these admissions, she had the removal of ureteral stents and the exchange of her percutaneous nephrostomy tubes, and she was discharged with the plan to continue isavuconazole. 18 months after the initial presentation, the patient completed a six-month course of isavuconazole after the last resumption of the medication without interruption; ureteral stents stayed for four months, nephrostomy tubes stayed for eight months, and kidney function stabilized with a baseline Cr of 1.4 mg/L and an eGFR of 49 ml/min.

## Discussion

Renal involvement in mucormycosis is a rare form of infection and often results from dissemination through hematogenous spread. Isolated renal mucormycosis (IRM) is even more uncommon and is associated with a very high mortality rate in cases of bilateral renal involvement. In the setting of negative blood cultures yet positive intraoperative ureteral cultures, we present a case of ascending mucormycosis [3].

The clinical presentation in our case aligns with that of previously reported cases, consisting of typical pyelonephritis symptoms such as fever and back pain for several weeks. The delay in diagnosis when these cases are mistaken for bacterial pyelonephritis may lead to additional complications, including AKI, fungemia, kidney infarction, or septic shock. Persistence of symptoms despite antibiotic treatment, along with risk factors such as diabetes, IV drug use, and immunodeficiency, are some of the clues for a possible urinary fungal infection [4]. Another shared characteristic of these cases is calyx-pelvic system dilatation and non-obstructive hydronephrosis. In this case, hydronephrosis prompted a cystoureteroscopy and ureteral culture, which were the only microbiological evidence of Rhizopus. Cultures from the kidney are typically needed to identify the organism since routine urine and blood cultures are often negative [5]. Our case demonstrates that a ureteral culture can be sufficient for an early, definitive diagnosis.

Historically, amphotericin B deoxycholate was considered the first-line treatment for mucormycosis. However, its use has been discouraged due to its high toxicity and lack of superiority compared to lipid-based drugs. Although liposomal amphotericin B is now the standard anti-fungal for Mucor/Rhizopus, it still carries a significant risk of nephrotoxicity, limiting its use [6]. The nephrotoxicity of amphotericin B is cumulative and worsens with a longer duration of use. Amphotericin B can cause acute toxicity, including hyperkalemia, which occurred in our patient. Treatment duration with amphotericin B in the reported studies varied widely from three weeks to three years, with an average duration of six months. Complete resolution of the immune defect or radiological resolution could help determine treatment duration [6,7].

There are many published cases of renal mucormycosis that were treated with some combination of bilateral or unilateral nephrectomy, amphotericin B, and posaconazole [8-11]. One systematic review analyzed 60 cases of renal mucormycosis and found that 60% were treated with surgery and anti-fungal medications, 12% were treated with surgery alone, and 13% were treated with anti-fungal therapy alone. Of these eight cases, only two were treated with isavuconazole due to its limited availability in low-income countries [12]. Patients with bilateral renal mucormycosis who do survive almost always undergo nephrectomy or surgical debridement, in addition to aggressive antifungal therapy with amphotericin B with or without posaconazole [5]. The first reported survival case of bilateral IRM occurred in 2004 in a patient treated with amphotericin B deoxycholate and bilateral nephrectomy, leaving the patient dialysis-dependent [13].

To the best of our knowledge, there has only been one case report of bilateral renal mucormycosis survival without surgical intervention, which was published by Marsh et al. in 2018. This patient had similar risk factors (e.g., insulin-dependent diabetes, uncontrolled diabetes, and substance abuse) and a similar presentation to our patient, and the ureteral culture was sufficient to reveal Rhizopus. The patient was successfully treated with two weeks of induction therapy with liposomal amphotericin B, followed by three months of oral posaconazole. Despite the use of amphotericin B, kidney function recovered, and dialysis was stopped [14]. Our case, therefore, represents the second reported case of bilateral IRM survival with antifungal treatment alone and the first case of successful treatment with isavuconazole.

Isavuconazole and posaconazole are novel triazoles that have activity against Mucor/Rhizopus and Aspergillus. The VITAL phase III clinical trial showed that isavuconazole has comparable efficacy to liposomal amphotericin B for the treatment of disseminated mucormycosis [15]. The US FDA approved isavuconazole as a first-line treatment for invasive mucormycosis in 2015. However, it is still considered a salvage treatment in Europe, and there is limited clinical data on the success of isavuconazole for the treatment of IRM. Isavuconazole is shown to be generally safe for the kidneys but can be hepatotoxic, which may limit its use.

Deciding on management for bilateral renal mucormycosis is complex due to the known nephrotoxicity of the preferred antifungal treatment, the potential for treatment failure, and the need for lifelong renal replacement therapy when bilateral nephrectomy is performed. In this case, amphotericin B was stopped after six days, and nephrectomy was deferred to preserve kidney function. A six-month course of isavuconazole was successful in treating infection, leading to survival while preserving renal function and avoiding long-term dialysis.

## Conclusions

In IRM, the kidneys face three detrimental factors: infection, nephrotoxic amphotericin B, and surgical debridement. In the setting of mild sepsis and hemodynamic stability, preserving kidney function could be an achievable treatment goal secondary to saving the patient’s life. In our case, amphotericin B was used only for six days. Therefore, isavuconazole was successful in controlling infection without nephrotoxicity or the need for surgical debridement. Kidney function remained stable, and our patient did not require dialysis long-term. The reason for a recurrent positive culture of nephrostomy tubes during subsequent admissions may be due to noncompliance with her antifungal regimen. Once our patient was adherent to treatment, the infection was completely treated. In our case, six months from the last resumption was sufficient to clear the infection.
